# Study on the Inhibitory Activity of a Synthetic Defensin Derived from Barley Endosperm against Common Food Spoilage Yeast

**DOI:** 10.3390/molecules26010165

**Published:** 2020-12-31

**Authors:** Laila N. Shwaiki, Aylin W. Sahin, Elke K. Arendt

**Affiliations:** 1 School of Food and Nutritional Sciences, University College Cork, T12K8AF Cork, Ireland; l.shwaiki@umail.ucc.ie (L.N.S.); aylin.sahin@ucc.ie (A.W.S.); 2 APC Microbiome Ireland, University College Cork, T12K8AF Cork, Ireland

**Keywords:** synthetic barley peptide, plant antimicrobial peptides, barley endosperm, spoilage yeast, defensin

## Abstract

In the food industry, food spoilage is a real issue that can lead to a significant amount of waste. Although current preservation techniques are being applied to reduce the occurrence of spoilage microorganisms, the problem persists. Food spoilage yeast are part of this dilemma, with common spoilers such as *Zygosaccharomyces*, *Kluyveromyces*, *Debaryomyces* and *Saccharomyces* frequently encountered. Antimicrobial peptides derived from plants have risen in popularity due to their ability to reduce spoilage. This study examines the potential application of a synthetic defensin peptide derived from barley endosperm. Its inhibitory effect against common spoilage yeasts, its mechanisms of action (membrane permeabilisation and overproduction of reactive oxygen species), and its stability in different conditions were characterised. The safety of the peptide was evaluated through a haemolysis and cytotoxicity assay, and no adverse effects were found. Both assays were performed to understand the effect of the peptide if it were to be consumed. Its ability to be degraded by a digestive enzyme was also examined for its safety. Finally, the peptide was successfully applied to different beverages and maintained the same inhibitory effects in apple juice as was observed in the antiyeast assays, providing further support for its application in food preservation.

## 1. Introduction

Plants are organisms with distinct mechanisms evolved to protect them from the harsh environments posed by nature. In some plants, the development of physical traits such as thorns and spines is a tool that can deter herbivores [1]. Amongst these complex mechanisms is the production of antimicrobial peptides (AMPs), which are known to challenge the presence of microbial and fungal infections. They are part of the innate immune system of a plant and can differ based on their structure and their selective nature [2]. Their cationic and cysteine-rich sequences make them effective agents against microbial and fungal cells, but less so towards mammalian cells due to their weaker affinity to neutrally/positively charged membranes [3]. Many AMPs are grouped together based on their conformation and sequence. Defensins are one class of plant AMPs that are characterised as cationic; they contain 45–54 amino acids and can form cysteine-stabilised alpha/beta motifs (CSαβ motif) [4]. Thionins, snakins/GASA, hevein- and knottin-type peptides, and cyclotides are other well-recognised plant AMPs known for their impact on plant defence systems [5]. 

Plant AMPs can be purified using various extraction methods that ensure their precipitation. These types of natural extraction methods have been developed for AMPs from a wide range of plants [6,7,8,9]. The chemical synthesis of pure peptides derived from known amino acid sequences is another preferred method of production [10]. Solid phase peptide synthesis (SPPS) and solution-phase synthesis (SPS) are both forms of chemical synthesis adapted for the production of these peptides. 

Microbial spoilage is a challenge faced by the food industry as it can lead to major economic losses and consequently to the waste of a significant amount of food. Spoilage microorganisms can range from bacterial to fungal pathogens. Yeast are sometimes overlooked as spoilers of food products; however, a large number of dairy, meat, fruit and vegetable products are effected by yeast species such as *Kluyveromyces*, *Debaryomyces*, *Zygosaccharomyces*, *Pichia*, *Candida*, *Yarowia* and *Saccharomyces* [11]. Plant AMPs have been explored for their potential as food preservatives in various studies, most of which deal with reducing infections in plants and crops caused by microbial and fungal pathogens [12,13,14]. This is significant to combat plant infections that can lead to major losses of agricultural crops. However, more studies need to be conducted on the application of plant AMPs for the preservation of food products. Food preservatives that deal with microbiological spoilage have already been established; the addition of chemicals, pasteurising or freezing foods, irradiation or modified atmospheric packaging are some examples [15]. Regardless of this, spoilage is still a recurring problem in the food industry, making it vital for more effective forms of preservatives to be discovered. Not only this, but consumer perception of the current forms of preservatives on the market has also become an influential driving factor for the development of more natural forms of preservatives [16].

This study will explore the antiyeast activity of a synthetic defensin peptide known as defensin-like protein 1 (D-lp1) (previously known as gamma-hordothionin). This sulphur-rich defensin is present in barley endosperm and has a known molecular weight mass of 5.25 kDa and a sequence length of 47 amino acids. It was first isolated by Mendez et al., 1990 and designated into the thionin family of plant AMPs [17]. Now, it is known that these peptides correspond to the characteristics of the family of plant defensins. In this study, D-lp1 was tested for its inhibitory effects against common food spoilage yeast. Its minimum inhibitory concentration (defined as the lowest concentration of peptide required to inhibit the visible growth of the yeast) and minimum fungicidal concentrations (the minimum concentration required to prevent yeast growth) [18] were determined. Its stability in different conditions and application in various beverages were investigated in order to establish its potential as a prospective preservative agent. Its safety and mechanism of action were also examined.

The lowest concentration of an antimicrobial that will inhibit the visible growth of a microorganism after overnight incubation was also identified.

## 2. Results

### 2.1. Secondary Structure Analysis of D-lp1

**Figure 1:**
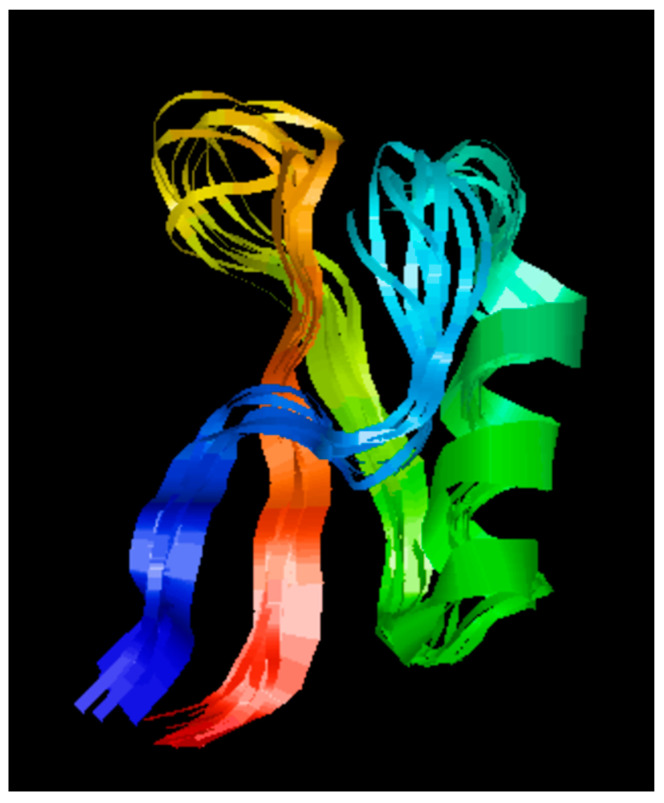
Secondary structure of defensin-like protein 1 (D-lp1) generated on RasMol demonstrating the triple stranded antiparallel beta-sheets and alpha helix structure present within the peptide.

The secondary structure of the peptide revealed triple-stranded antiparallel beta-sheets, an alpha helix, and corresponding connecting loops (Figure 1). 

### 2.2. D-lp1 Antiyeast Activity 

The antiyeast activity determined the minimum inhibitory concentration (MIC) or minimum fungicidal concentration (MFC) of the peptide for the five yeast. 

*Zygosaccharomyces bailii* and *Debaryomyces hansenii* were the most sensitive yeasts with MFC ranges of 50–100 μg/mL. *Saccharomyces cerevisiae* and *Zygosaccharomyces rouxii* were only inhibited at the highest concentration of 400 μg/mL (MFC and MIC range of 200−400 μg/mL, respectively). The fungicidal activity of the peptide was observed at all inhibitory concentrations against *Z. bailii* and *S. cerevisiae*, while only at 200 and 400 μg/mL against *D. hansenii* (Table 1). The observed fungicidal effects helped to distinguish between the inhibitory mechanism of the peptide (MIC) and its killing mechanism (MFC). No inhibition against *Kluyveromyces lactis* was detected.

### 2.3. Colony Count Assay

**Figure 2:**
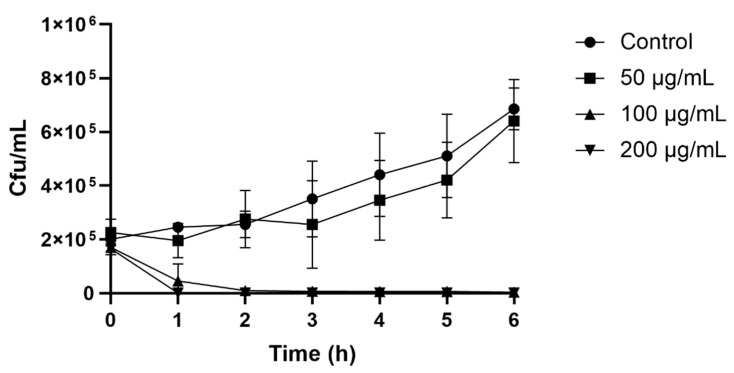
The time course required for D-lp1 to cause inhibition on *Z. bailii* over a period of 6 h. Peptide concentrations of 50, 100, and 200 µg/mL were tested alongside a control.

The colony count assay observed the time course of yeast inhibition as a result of the peptide’s antiyeast activity. After only 2 h of incubation, 100 (MIC) and 200 μg/mL of peptide resulted in a rapid decrease in cell numbers. The control with no peptide and a peptide concentration of 50 μg/mL showed a steady increase in *Z. bailii* growth, whose cell growth after 6 h reached 6.3 × 10^5^ and 5.3 × 10^5^ cfu/mL, respectively (Figure 2). 

### 2.4. Peptide Stability

The peptide’s stability in concentrated salt solutions, different pH and high heat was performed to reveal its potential to maintain its antiyeast activity under these conditions.

**Figure 3:**
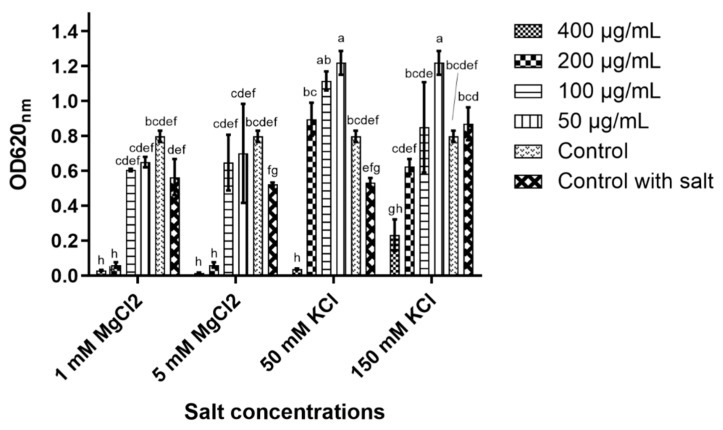
The effect of different salt concentrations (MgCl_2_ and KCl) on the antiyeast activity of the peptide. ANOVA analysis was performed to determine the significant differences between the peptide’s inhibitory activity in these salt concentrations (at various concentrations of the peptide). The values containing different letters vary statistically (a–h).

In 5 and 50 mM magnesium chloride (MgCl_2_), the peptide’s antiyeast activity was only affected at 100 μg/mL, while at the higher concentrations (200 and 400 μg/mL), nearly full inhibition was detected (Figure 3). D-lp1 was affected more in potassium chloride (KCl) as 400 μg/mL was the only concentration observed to cause inhibition in the 50 mM KCl solution. In 150 mM KCl, some inhibition occurred at 400 μg/mL; however, growth was still evident. ANOVA determined significant differences in the peptide’s ability to cause yeast inhibition in the different salt concentrations (at the various peptide concentrations tested) (Figure 3).

Applying thermal treatment at 100 °C for 15 min did not change the activity of the peptide. Full inhibition was observed on the growth of *Z. bailii* at concentrations up to the MIC (100 µg/mL).

Modifying the media to the different pH of 3, 5 and 7 resulted in no change in the peptide’s inhibitory activity. Full inhibition of the yeast was detected at the concentrations tested (200 and 100 μg/mL) due to the retention of its antiyeast activity. At pH 9 and 11, no yeast growth was seen in the controls.

### 2.5. Mechanism of Action 

**Figure 4:**
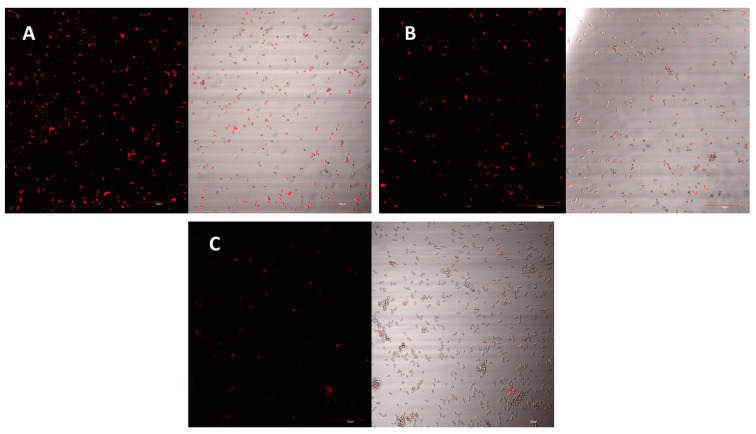
*Z. bailii* membrane permeabilisation observed under the confocal laser scanning microscope (CLSM) as a result of the peptide at concentrations of (**A**) 400 µg/mL, (**B**) 200 µg/mL and (**C**) 100 µg/mL.

**Figure 5:**
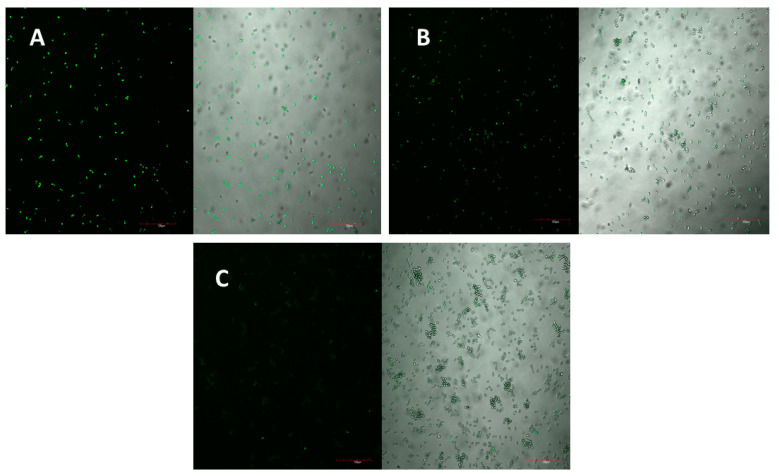
The overproduction of reactive oxygen species (ROS) generated by the presence of the peptide at the difference concentrations of (**A**) 400 µg/mL, (**B**) 200 µg/mL and (**C**) 100 µg/mL. A reduction in ROS overproduction is observed as the concentration of D-lp1 is reduced.

The peptide’s mechanism of action was studied using laser scanning microscopy. The peptide was observed to cause both membrane permeabilisation and overproduction of reactive oxygen species (ROS) in a dose-dependent manner. At the highest concentration of D-lp1 (400 μg/mL), complete permeabilization of the yeast cells was observed, with the level of permeabilisation decreasing as the peptide concentration was reduced (200 and 100 μg/mL) (Figure 4A–C). A similar effect was observed for the overproduction of ROS, with greater ROS detected at the higher concentration of 400 μg/mL (Figure 5A–C). 

### 2.6. Total Nucleotide Leakage

**Figure 6:**
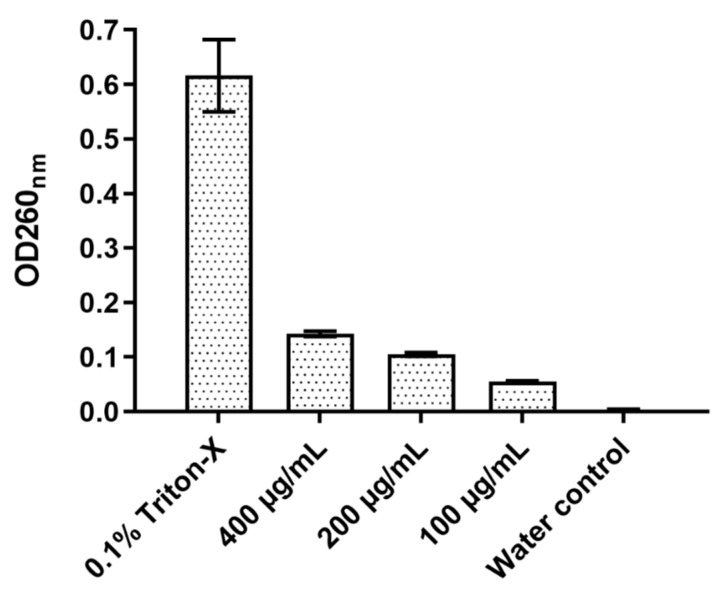
The total nucleotide leakage detected from *Z. bailii* 6 h after inoculation and incubations with the peptide at different concentrations.

The effect of D-lp1 on the cell membrane of *Z. bailli* was examined through a total nucleotide leakage assay (Figure 6). After a 4 h incubation period, an OD of 0.142 was measured at 400 µg/mL, a considerably higher OD compared to the negative control of *Z. bailii* with just distilled water (OD 0.003). The level of nucleotide leakage correlated with peptide concentration as 100 and 200 µg/mL resulted in ODs of 0.104 and 0.055, respectively.

### 2.7. Haemolytic Assay

The haemolysis of red blood cells was used as an indicator for the safety of the peptide. D-lp1 was not found to cause haemolysis even at the highest concentration of 400 µg/mL, with less than 10% haemolysis observed.

### 2.8. Cytotoxicity Assay

**Figure 7:**
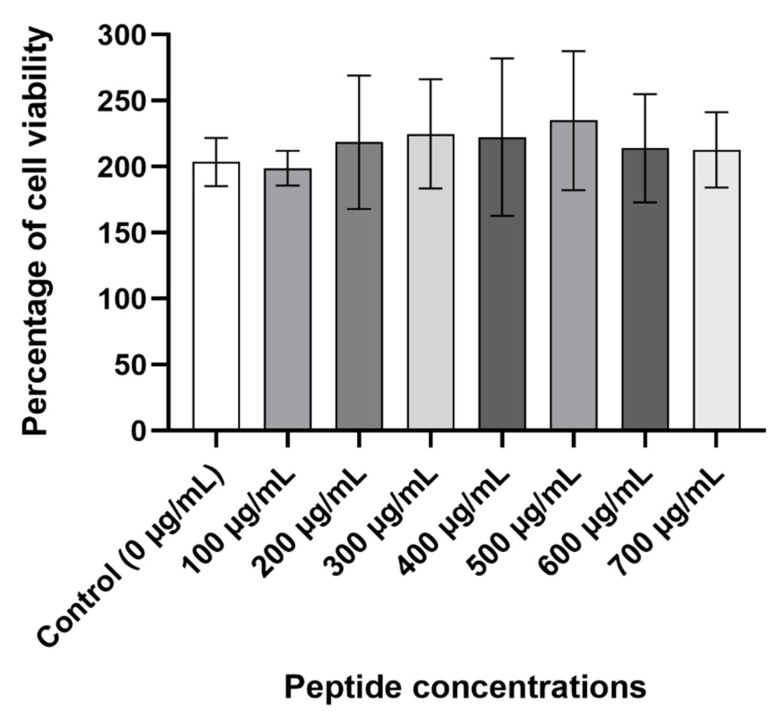
Percentage of cell viability caused by the different concentrations of peptide (from 100 to 700 µg/mL).

The viability of Caco-2 cells was measured to further determine the peptide’s safety. At all the concentrations that were tested, D-lp1 did not cause a reduction in the viability of the cells compared to the control cells containing only water (Figure 7). 

### 2.9. Resistance to Proteolytic Digestion

D-lp1 was tested for its susceptibility to proteolytic digestion with the aim of investigating its safety as a preservative. Alpha-chymotrypsin was used as the proteolytic enzyme in order to mimic the environment commonly found in the gut. At all molar concentrations, the enzyme was capable of digesting the peptide. Yeast growth was observed at all concentrations of peptide tested (400–100 µg/mL), indicating a reduction in its antiyeast activity.

### 2.10. Peptide Activity in Different Beverages

D-lp1 was tested for its inhibitory effect against *Z. bailii* in apple juice, Fanta Orange and wine. In the apple juice, the peptide was fully effective at inhibiting *Z. bailli* at the known inhibitory concentrations (400, 200 and 100 µg/mL). In the Fanta Orange, only 200 and 400 µg/mL caused a reduction in yeast growth, while in the wine, only partial inhibition (at 400 µg/mL) was observed. 

## 3. Discussion

The growing demand for natural preservatives has made plant-derived additives a popular alternative to the current chemical preservatives that consumers may find unappetising [19]. In recent years, plant AMPs have risen in popularity for their application to counteract microbial pathogens. Due to the laborious processes involved in the purification of these peptides from plants, the chemical synthesis of AMPs can be a direct alternative. The current study examined the effect of a synthetic peptide derived from the barley endosperm, referred to here as D-lp1, on the growth of common food spoilage yeast. The peptide’s amino acid sequence was previously discovered by Mendez et al., 1990 [17]. Their work on its structure and characterisation led them to categorise the peptide into the thionin family, a family of potent AMPs whose structures are not dissimilar to plant defensins. Previously known as gamma-purothionin, D-lp1′s 47 amino acid distribution within its sequence renders the peptide a structure homologous to plant defensins. The structure obtained from the RasMol programme illustrated the triple-stranded antiparallel beta-sheets and alpha helix present there. A cysteine-stabilised alpha-helical motif is formed by these beta and alpha helix structures which are being held together by three disulphide bridges in the hydrophobic core [19]. These characteristics are common amongst peptides in the family of plant defensins.

The peptide’s high net charge (+9), the presence of eight cysteine residues, and its basic nature can be used to explain its strong antiyeast activity [20]. Four of the five yeast tested were sensitive to the peptide, with MIC ranges as low as 50–100 µg/mL observed for *Z. bailii* and *D. hansenii.* The high net charge and rich presence of cysteine residues are common characteristics found in AMPs with potent antimicrobial activity [20,21,22]. The eight arginine residues present in D-lp1′s amino acid sequence contribute to the cationicity of the peptide, which permits easier interaction with the negatively charged yeast membrane, resulting in its disruption and cell death [22]. 

The peptide’s stability in the MgCl_2_ concentrations revealed minimal changes to its antiyeast activity, while in 150 mM KCl, a reduction in its inhibitory activity was apparent. This effect could be hypothesised to have occurred due to the presence of K+ cations causing a reduction in the net charge of the yeast cell wall. This reduction in net charge could have caused the peptide to be repulsed from the yeast cell [23]. The shift to a more negatively charged yeast cell could have also caused the overall charge of the peptide to be modified. As a consequence, this could have triggered structural changes to the peptide that may explain the reduced antiyeast activity detected in the presence of KCl [24]. Testing the stability of D-lp1 in the salts gives an insight on how the peptide might react in high salt environments frequently found to harbour spoilage yeast such as *Z. bailli*. Modifying the media to pH 3, 5 and 7 did not alter the peptide’s antiyeast activity, while in the higher pH (9 and 11), no yeast growth was observed in the controls. This was likely due to the unsuitable growth conditions for the yeast, rather than the effect of the peptide [25]. It has been stated that the biological function of a peptide can be altered in a solution that holds a pH equal to a peptide’s isoelectric point (pI) [26]. The high pI of D-lp1 (9.9) may have resulted in the retention of its antiyeast activity this range of pH (pH 3, 5 and 7). Thermal treatment also resulted in its normal antiyeast activity, further supporting its potential for application as a preservative. The peptide’s robust tertiary structure resulting from the various disulphide bonds formed by the eight cysteine residues present in its sequence could explain this stability [27].

Both mechanisms of actions examined were found to be dose dependant, with the highest concentration of 400 µg/mL causing the most permeabilisation and overproduction of ROS. The cationic nature of the peptide causing an interaction with the negatively charged yeast membrane could explain the high levels of permeabilisation that were detected [28,29]. This mechanism of action has been widely studied in various plant AMPs [30,31,32,33]. The total nucleotide leakage assay presents further evidence of the rate of membrane permeabilisation instigated by the peptide. The damage caused to the yeast membrane could have resulted in the nucleotide leakage from the cells [34,35]. The peptide’s impact on the yeast membrane can also lead to a cascade of reactions, including the overproduction of ROS [36]. The generation of these species by yeast cells plays a role in normal cell function; however, the introduction of a stress factor like D-lp1 can ultimately lead to the overproduction of ROS, and subsequently to cell death [37]. 

The peptide’s safety was evaluated for its intended use as a potential preservative. Its haemolytic and cytotoxicity activity were studied, giving an indication of its safety if it were to be consumed. The haemolytic activity of a peptide can generally be linked to its cytotoxicity and to its ability to cause harmful effects on eukaryotic cells [38]. In the presence of D-lp1, the percentage of ruptured red blood cells measured was minimal, with less than 10% haemolysis observed at the highest concentration of 400 µg/mL. This low percentage of haemolysis signifies the peptide’s safety and its suitability as a potential preservative. The cytotoxicity assays revealed a lack of toxicity towards the Caco-2 cells, further supporting the peptide’s safety. This attribute is common amongst cationic AMPs whose affinity for negatively charged membranes makes them more attracted to the membranes of microbial cells than those of eukaryotic cells (that are predominantly composed of neutrally and/or slightly positively charged membranes) [39]. In addition, the presence of cholesterol in eukaryotic cell membranes can act as a rigid barrier to the lipid bilayer structure of the cell that can impede the destruction of the membrane via the action of cationic AMPs, not unlike D-lp1 [40]. 

D-lp1′s sensitivity to α-chymotrypsin, a proteolytic digestive enzyme, was also examined to better characterise its safety if consumed. In the gastrointestinal tract, the presence of various digestive enzymes would make it an ideal environment for the breakdown of the peptide. D-lp1 was found to be sensitive to the α-chymotrypsin at all molar concentrations tested, providing additional evidence for its safety. 

As a proof of concept, D-lp1 was applied to the different beverages. The peptide was found to retain its full antiyeast activity in the apple juice, while in the Fanta Orange and wine, the higher concentrations still caused inhibition. The consistency of beverages such as these makes it easier for the peptide to perform its inhibitory effects on the yeast. This matrix creates a suitable environment for the peptide to impart its effect on the cell membrane of the yeast, made possible by the consistency of the beverages. Nisin, an AMP that is known for its potent antimicrobial activity and by its successful incorporation into various food products, has been applied in beverages with similar consistencies. Its application in alcoholic beverages has been found to inhibit the growth of common beer and wine microbial food spoilers [41]. The successful incorporation of the peptide into the beverages tested in this study helps to demonstrate its potential as a novel preservative agent.

## 4. Materials and Methods

### 4.1. Defensin-Like Protein 1 (D-lp1) Synthesis 

D-lp1, a 47 amino acid sequence peptide derived from barley (*Hordeum vulgare*) endosperm, was chemically synthesised by GL Biochem (manufacture, Shangai, China) Ldt to a purity of 80% as indicated by the supplier. The peptide was resuspended in ultrapure water at a concentration of 2 mg/mL. The amino acid sequence of D-lp1 can be seen in Table 2.

### 4.2. D-lp1 Secondary Structure 

The secondary structure of the peptide was developed on the programme RasMol [42] using the amino acid sequence obtained from the Protein Data Bank [43]. The structure of the peptide was reviewed using the model built from the programme. 

### 4.3. Yeast Strains

The different yeast strains used throughout the study were as follows: *Zygosaccharomyces bailli* Sa 1403, *Zygosaccharomyces rouxii* ATCC14679, *Kluyveromyces lactis* ATCC 56498, *Debaryomyces hansenii* CBS 2334 (DMSZ (Braunschweig, Germany)), and *Saccharomyces cerevisiae* Baker’s yeast (Puratos, Belgium). Each yeast was grown aerobically in Sabouraud dextrose (SD; Sigma Aldrich, St. Louis, MO, USA) agar at 25 °C. Overnight cultures of the yeast were prepared in SD broth (pH 5.3) at the same temperature under gentle agitation at 130 rpm. All media and reagents used were obtained from Sigma-Aldrich (St. Louis, MO, USA), unless otherwise stated.

### 4.4. Determination of D-lp1 Antiyeast Activity

The MIC of D-lp1 against the 5 yeast strains was also examined as described by Shwaiki, Arendt & Lynch, 2020 [44]. The assay was performed in compliance with the method outlined by the National Committee for Clinical Laboratory Standards (NCCLS M27A- [45]. Briefly, a 10^4^ cfu/mL solution of each yeast strain was prepared from overnight cultures in SD broth and transferred into the wells of a flat-bottom 96-well microtitre plate (Sarsdedt, Nümbrecht, Germany). The peptide was added into the first well and serially diluted to concentrations ranging from 12.5 to 400 μg/mL. A positive control of just distilled water with no peptide was also included. The plates were incubated for 48 h at 25 °C in a microtitre plate reader (Multiskan FC Microplate Photometer, Thermo Scientific, Waltham, MA, USA) under gentle agitation. The optical density at 620 nm was measured every 2 h against broth as a blank.

The fungistatic and fungicidal activity of the peptide against the susceptible yeast was also examined to determine its MFC as described by Shwaiki, Arendt and Lynch, 2020 [44]. One hundred microlitres of each yeast suspension from an antiyeast assay was spotted onto SD agar plates and incubated for 48−72 h, depending on the yeast. *D. hansenii*, *S. cerevisiae* and *Z. bailii* were incubated for 48 h while *Z. rouxii* and *K. lactis* were incubated for 72 h.

### 4.5. Colony Count Assay

A colony count assay was performed as described by Shwaiki, Arendt and Lynch, 2020 [44]. The time required for the peptide to influence yeast growth was observed. Briefly, a yeast suspension of 10^5^ cfu/mL was inoculated with different concentrations of D-lp1 (50, 100 and 200 µg/mL) and incubated for a period of 6 h. At 1 h intervals, 100 µL of the suspensions was spread onto SD agar plates and subsequently incubated for 48 h at 25 °C. The control consisted of a yeast suspension with water instead of peptide. 

### 4.6. Peptide Stability

D-lp1 was tested for its stability so as to determine whether it can retain its antiyeast activity in different environmental conditions which may be encountered in food products. High salt, heat and a range of pH were used to test this. The indicator yeast *Z. bailii* was treated due to its sensitivity towards the peptide. Concentrations of 50, 100 and 200 µg/mL of the peptide were tested.

Salt solutions of 1 and 5 mM MgCl_2_ and 50 and 150 mM KCl were prepared and added to SD broth. An antiyeast assay was executed using the modified broth. Controls of modified broth, yeast and no peptide were also included.

To determine the effect of thermal treatment, the peptide was heated for 15 min at 100 °C and left to cool before an antiyeast assay was performed.

The effect of different pH on the peptide was conducted by changing the pH of the SD media used to perform the antiyeast assay. This was done to examine the peptide’s ability to retain its antiyeast activity in various pH conditions that may be encountered if the peptide was to be applied as a food preservative. pH 3, 5, 7, 9 and 11 were tested by modifying the media with 0.1 M hydrochloric acid and 1 M sodium hydroxide to lower or increase the pH, respectively. Controls of media modified to the different pH containing the yeast and no peptide were used.

### 4.7. Membrane Permeabilisation

One potential mechanism of action of the peptides against the yeast is their ability to permeabilise yeast membrane. This mechanism is detected using propidium iodide, a dye that when inserted through the permeabilised yeast membrane, can bind and stain nucleic acids. The assay was performed as described by Shwaiki et al., 2020 [46]. Briefly, a *Z. bailii* cell suspension of 10^6^ cfu/mL in SD broth was incubated for 2 h with different peptide concentrations ranging from 100 to 400 µg/mL. A final concentration of 5 µM propidium iodide was added and incubated for 20 min at room temperature in dark conditions. After washing with SD broth, this solution was then viewed under a confocal laser scanning microscope (CLSM) (Olympus FV1000, incorporating an IX81 inverted microscope, Germany) using maximal excitation (λEx) and maximum emission (λEm) wavelengths of 535 nm and 617 nm, respectively. Triton X-100 (0.1%) and water were used at the positive and negative controls, respectively.

### 4.8. Overproduction of Reactive Oxygen Species (ROS)

The peptides’ ability to cause an overproduction of ROS in the yeast was assessed using the protocol described by Shwaiki, Arendt and Lynch, 2020 [44]. Briefly, a yeast suspension of 10^6^ cfu/mL was prepared from an overnight solution of *Z. bailii.* Dihydrorhodamine 123 (5 µg/mL) was incubated with this solution at 25 °C for 2 h to facilitate its uptake into the cells and, subsequently, its oxidation to rhodamine 123 in the presence of ROS. Following this, the sample was centrifuged at 3500× *g* for 5 min, and the cells were washed with SD broth. Different concentrations of peptide were added (100, 200 and 400 µg/mL) and incubated at 25 °C for 1 h. After incubation, 0.6 M potassium chloride was used to wash the cells once more before measuring the fluorescence under the CLSM at maximal excitation (λEx) and maximum emission (λEm) wavelengths of 488 and 538 nm, respectively. Two millimetres of hydrogen peroxide (H_2_O_2_) and water were used as positive and negative controls, respectively.

### 4.9. Total Nucleotide Leakage

The total nucleotide leakage of *Z. bailii* resulting from the peptide’s activity was analysed according to the protocol by Li et al., 2016 [35], with some modifications. An overnight culture of *Z. bailli* was used to prepare a 10^6^ cfu/mL suspension that was washed twice using phosphate saline buffer (PBS). D-lp1 was added to this yeast suspension at concentrations of 100, 200 and 400 μg/mL and incubated for 4 h at 25 °C, after which the yeast cells were removed via filtration through a 0.22 μm filter. The OD at 260 nm was measured for each concentration. A positive and negative control of 0.1% Triton X-100 and water were used, respectively.

### 4.10. Haemolytic Activity

D-lp1′s ability to rupture red blood cells and test their safety with regards to their application in food was evaluated. The assay was performed as described by Thery & Arendt, 2018 [47]. Fresh sheep’s blood (Oxoid™) was washed three times with equal volumes of phosphate-buffered saline (PBS). A 10 mL 4% solution was prepared using PBS and then transferred into 1.5 mL Eppendorf tubes, in conjunction with 100, 200 and 400 µg/mL of the peptide. After incubation at 37 °C for 1 h, the sample was centrifuged, and the supernatant transferred into a 96-well plate to measure its optical density at a wavelength of 405 nm. Triton X-100 (0.1%) and PBS were used as positive and negative controls, respectively. The percentage of haemolysis was calculated using the formula below.

$$\%\;\mathrm{Haemolysis}\;=\;\frac{\left(A405\;\mathrm{peptide}\;\mathrm{treatment}\right)-\left(A405\mathrm{PBS}\right)}{\left(A405\;0.1\%\;\mathrm{Triton}\;X-100\right)-\left(A405\;\mathrm{PBS}\right)}$$

### 4.11. Cytotoxicity Assay

The ability of a peptide to cause a harmful or toxic effect on human cells is referred to as its cytotoxicity activity [48] A cytotoxicity assay was performed as described by Shwaiki, Arendt and Lynch, 2020 using a colonic cell line [44]. Briefly, Caco-2 cells (ECACC) were prepared in Dulbecco’s modified Eagle media (DMEM) that had been supplemented with 1% nonessential amino acids and 10% foetal bovine serum (FBS). A cell suspension of 1 × 10^5^ cells/mL was prepared, 200 µL of which was added into the wells of a flat-bottom 96 well microtitre plate and incubated for 24 h at 37 °C with 5% CO_2_. Following this incubation, the media were removed and D-lp1 was added into the wells at concentrations ranging from 100 to 700 μg/mL, together with DMEM supplemented with 2.5% FBS. Each well contained a final volume of 200 μL. The controls consisted of sterile water in DMEM. The plate was incubated at 37 °C for 24 h. The media in the wells was discarded and 100 μL of DMEM and 10 μL of the MTT labelling reagents (Cell proliferation Kit I MTT; Sigma, Ireland) were transferred to each well. Following a 4 h incubation period, a solubilisation buffer (100 μL) was added, and the plate was incubated for a further 24 h. A fluorometric spectrophotometer was used to measure the cell viability in each well at a wavelength of 570 nm using a background reading of 690 nm.

### 4.12. Resistance to Proteolytic Degradation

This assay was performed following the protocol described by Shwaiki et al., 2020 to characterise the peptide’s ability to withstand proteolytic degradation [46]. This was done to simulate what the peptide may encounter if applied as a food preservative. In brief, the digestive enzyme α-chymotrypsin was incubated with the peptide at different concentrations: enzyme molar ratios of 60:1, 250:1, and 2500:1 for 4 h at 37 °C. This was followed by inactivation at 80 °C for 10 min. An antiyeast assay was then performed to test the peptide at different concentrations (50, 100, 200 and 400 µg/mL) against *Z. bailii*.

### 4.13. Application of D-lp1 in Different Food Matrices

The application of D-lp1 was assessed in different food matrices against *Z. bailii*, the most susceptible yeast towards the peptide.

The high sugar or salt content found in soft drinks and fruit juices make it a favourable environment for *Z. bailii* to grow [49]. The antiyeast activity of the peptide was tested in apple juice, pH 3.2 (*Tropicana Apple*, pressed apple); Fanta Orange, pH 3.1 (*Coca-Cola*, Ireland); and wine (*Faber*, Chardonnay), pH 3.05. D-lp1 was tested via the microtiter plate method using a 10^4^ cfu/mL suspension of yeast made up in filter sterilised solutions of the beverages. The peptide was tested at concentrations ranging from 50 to 400 µg/mL, and its antiyeast activity was observed over 48 h by measuring the optical density at 620 nm every 2 h against a sample of each beverage as the blanks. Controls consisting of the beverages inoculated with the same concentration of yeast without the peptide were also included.

### 4.14. Statistical Analysis

To determine the significant difference between the results obtained for the salt stability assay, analysis of variance with ANOVA (SigmaStat, SPSS Inc., Chicago, IL, USA), was performed. The statistical significance between the inhibitory properties observed in the different salt concentrations (at various peptide concentrations) was analysed. A probability of *p* < 0.05 was considered statistically significant.

## 5. Conclusions

This study illustrates the potential of a synthetic peptide derived from barley endosperm to act as a novel food preservative. Consumers’ opinions of the current forms of preservatives stems from the perception that foods should be more natural and unaffected by the addition of chemical preservatives. Taking advantage of plants and the AMPs that they produce may be an effective solution to this. Naturally extracting AMPs straight from the plant can be time consuming and costly. The current cost of synthesis for the production of peptides such as the one presented in this study can limit their application in food. The production of a peptide with similar length and amino acid composition as D-lp1 can amount to on average USD 10 per amino acid for a peptide of up to 4mg/mL and with a purity of >85% (price is based on general rates within the market of peptide synthesis). Although this cost of synthesis may be a disadvantage now, with time, the cost of synthesis will reduce, making it more feasible to synthesise peptides. In addition to this, the time-effective production process required to produce synthetic AMPs and the potential to develop a peptide of high purity could make this the preferred method of production. The approach presented in this study, although not currently feasible for wide-scale production, demonstrates a proof of principle for the application of synthetic AMPs in food preservation.

## Figures and Tables

**Table 1:** Minimum inhibitory concentration (MIC) and minimum fungicidal concentration (MFC) of the peptide against the five yeast.

Yeast	Minimum Inhibitory Activity (MIC and MFC Ranges)
*Zygosaccharomyces bailii*	50−100 μg/mL (MFC)
*Zygosaccharomyces rouxii*	200−400 μg/mL (MIC)
*Kluyveromyces lactis*	No inhibition
*Saccharomyces cerevisiae*	200−400 μg/mL (MFC)
*Debaryomyces hansenii*	50−100 μg/mL (MFC at higher concentrations)

**Table 2:** D-lp1 amino acid sequence.

*D-lp1 Amino Acid Sequence*
RICRRRSAGFKGPCVSNKNCAQVCMQEGWGGGNCDGPLRRCKCMRRC

## Data Availability

The data presented in this study are available on request from the corresponding author.
